# Neutrophil and Monocyte Function in Patients with Chronic Hepatitis C Undergoing Antiviral Therapy with Regimens Containing Protease Inhibitors with and without Interferon

**DOI:** 10.1371/journal.pone.0166631

**Published:** 2016-11-18

**Authors:** Martina Gambato, Noelia Caro-Pérez, Patricia González, Nuria Cañete, Zoe Mariño, Sabela Lens, Martín Bonacci, Concepció Bartres, José-María Sánchez-Tapias, José A. Carrión, Xavier Forns, Manel Juan, Sofía Pérez-del-Pulgar, María-Carlota Londoño

**Affiliations:** 1 Liver Unit, IDIBAPS and CIBEREHD, Hospital Clínic Barcelona, Barcelona, Spain; 2 Liver Unit, Gastroenterology Department, Hospital del Mar, IMIM (Hospital del Mar Medical Research Institute), Barcelona, Spain; 3 Servei d’Immunologia, Hospital Clínic Barcelona, Barcelona, Spain; University of Sydney, AUSTRALIA

## Abstract

Real-life data showed an increased incidence of bacterial infections in patients with advanced liver disease receiving a protease inhibitor (PI)-containing antiviral regimen against hepatitis C (HCV). However, the causes of this event are unknown. We hypothesized that PIs might impair innate immune responses through the inhibition of proteases participating in the anti-bacterial functions of neutrophils and monocytes. The aims of the study were to assess phagocytic and oxidative burst capacity in neutrophils and monocytes obtained from patients receiving a PI containing-antiviral regimen, and to determine cytokine secretion after neutrophil stimulation with flagellin. Forty patients with chronic HCV (80% with cirrhosis) were enrolled in the study, 28 received triple therapy (Group A) with pegylated-interferon and ribavirin for 4 weeks followed by the addition of a PI (telaprevir, boceprevir or simeprevir), and 12 patients received an interferon-free regimen (Group B) with simeprevir and sofosbuvir. Phagocytosis and oxidative burst capacity were analyzed by flow cytometry at baseline, week 4, and week 8 of therapy. In neutrophils from Group A patients, oxidative burst rate and oxidative enzymatic activity per cell significantly decreased throughout the study period (p = 0.014 and p = 0.010, respectively). Pairwise comparisons showed a decrease between baseline and week 4 and 8 of therapy. No differences were observed after the introduction of the PI. The oxidative enzymatic activity per cell in monocytes significantly decrease during the study period (p = 0.042) due to a decrease from baseline to week 8 of therapy (p = 0.037) in patients from Group A. None of these findings were observed in Group B patients. Cytokine secretion did not significantly change during the study in both groups. In conclusion, our data suggest that the use interferon (rather than the PI) has a deleterious effect on neutrophil and monocyte phagocytic and oxidative burst capacity in this cohort of patients with HCV-related advanced liver fibrosis.

## Introduction

The use of triple therapy (TT), which combines a first generation protease inhibitor (PI) with pegylated interferon (PegIFN) and ribavirin (RBV), was a major breakthrough in the treatment of hepatitis C due to a significant increase in the chances of achieving sustained virological response (SVR) [1–5]. However, the use of triple therapy (TT) was associated with an important increase in the number of treatment-related adverse events (including infections, clinical decompensation and death) in cirrhotic patients, especially in those with signs of portal hypertension (platelet count <100,000 mm^3^) or liver dysfunction (albumin levels < 35g/L) [6–8]. Moreover, in a recently published study by our group [9], patients with cirrhosis who received TT presented a significantly higher number of bacterial infections as compared to cirrhotic patients treated with PegIFN and RBV (25% vs. 9%; p = 0.001). We also found that the use of TT changed the pattern of infections with an increase in the number of respiratory tract infections (particularly with gram-positive cocci) in the group of patients treated with this combination. The latter differs from the typical infections observed in cirrhotic patients, which are spontaneous bacterial peritonitis or spontaneous bacteremia caused by gram-negative bacilli. More recently, data from the TARGET cohort evaluating the efficacy and safety of an IFN-free regimen (sofosbuvir [SOF] and simeprevir [SMV]) in liver transplant recipients with advanced hepatitis C recurrence evidenced a non-negligible rate of bacterial infections of 14.6% [10].

It is well known that patients with cirrhosis are at higher risk of developing bacterial infections. As described in detail previously [9], there are several mechanisms to explain the increased risk of infections in these patients including liver dysfunction, bacterial translocation, shunting, dysbiosis, immune dysfunction, and polymorphisms in *NOD2* or *TLR2* [11]. However, the change in the pattern of infections observed in patients receiving antiviral therapy with a PI, prompted us to study other mechanisms. A possible explanation and the basis of our hypothesis was that PIs might compromise the functions of different components of the innate immune system. The efficiency of bacterial elimination depends on the rapid recruitment of neutrophils from the circulation into the site of infection, phagocytosis and destruction of the microbe [12]. Similarly, monocytes/macrophages are essential in the initial host reaction to infection by initiating an inflammatory response. The activation of monocytes/macrophages is triggered by self and non-self recognition through specialized proteins expressed in cell membrane including MHC (major histocompatibility complex) and TLR (toll-like receptors) [13]. This recognition activates an intracellular cascade that leads to the production of cytokines and chemokines.

Protease inhibitors against hepatitis C might impair the function of the innate immune system by different mechanisms, such as blocking proteases/enzymes contained in neutrophils and monocytes (which are crucial to microbe destruction). Indeed, an *in vitro* study using various anti-HIV PIs showed a significant decrease in neutrophil functions including phagocytosis, superoxide production and chemotaxis, and neutrophil apoptosis [14]. A recently published study observed a decrease in neutrophils’ phagocytic capacity in patients receiving a PI-containing antiviral regimen as compared to patients treated with dual therapy with PegIFN and RBV [15]. Therefore, the aim of this study was to evaluate innate immune system responses to infections assessing phagocytosis, oxidative burst capacity and cytokine production in neutrophils and monocytes from patients with chronic hepatitis C undergoing antiviral therapy with a PI-containing regimen with and without IFN.

## Patients and Methods

### Patients

Between February 2014 and March 2015, 40, genotype 1-infected patients with chronic hepatitis C who completed at least 8 weeks of antiviral therapy with a first generation PI (with or without PegIFN and RBV) were included in this study. Patients were sequentially enrolled and received an IFN-based TT (Group A) or an IFN-free therapy (Group B), as explained below. Fibrosis stage was determined by transient elastography (F2: 7.6–9.4 kPa, F3: 9.5–14 kPa and F4: ≥14 kPa). In patients with fibrosis stage F4, an upper endoscopy and a hepatic venous gradient pressure (HVPG) measurement were performed before treatment in order to determine the presence of portal hypertension. Demographic, clinical and virological characteristics of patients enrolled in Group A (n = 28) or Group B (n = 12), are depicted in Table 1. A control group of 10 healthy individuals was also analyzed.

**Table 1:** Demographic, clinical and virological characteristics of the patients included in the study.

Characteristics(n = 40)	IFN-based triple therapy (PegIFN/RBV+TVR/BOC/SMV) (n = 28)	IFN-free therapy (SOF/SMV+RBV) (n = 12)	*p* value
Age (years)	59 (37–71)	57 (43–77)	0.998
Liver disease stage; n (%)			0.591
• F0-1	0	1 (8%)	
• F2-3	7 (25%)	0	
• F4	21 (75%	11 (92%)	
Previous IFN-based treatment; n (%)	16 (76%)	5 (42%)	0.290
Response to previous treatment; n (%)			0.227
• Non-Responder	11 (56%)	4 (80%)	
• Relapser	7 (44%)	1 (20%)	
IL28B Polymorphism; n (%)			0.679
• CC	5 (18%)	2 (17%)	
• CT	13 (46%)	7 (58%)	
• TT	3 (11%)	2 (17%)	
• Unknown	7 (25%	1 (8%)	
Protease inhibitor; n (%)			NA
• Telaprevir	10 (36%	-	
• Boceprevir	2 (7%)	-	
• Simeprevir	16 (57%)	12 (100%)	
Neutrophils (x 10^3^ cells/mm^3^)	2.9 (1.5–5.3)	2.6 (1.2–4.2)	0.688
Monocytes (x 10^3^ cells/mm^3^)	0.4 (0.2–0.8)	0.4 (0.2–0.9)	0.745
Platelets (x 10^3^ cells x mm^3^)	137 (68–204)	92 (34–393)	0.016
• >100.000	4 (17%)	8 (67%)	0.006
Total bilirubin (mg/dL))	0.8 (0.4–1.8)	0.9 (0.3–2.1)	0.344
AST (IU/mL)	77 (36–194)	89 (51–170)	0.420
ALT (IU/mL)	115 (45–221)	79 (43–155)	0.184
Albumin (g/L)	44 (38–50)	43 (34–47)	0.294
• < 35 g/L	0	1 (8%)	0.343
INR	1.1 (0.9–1.2)	1.2 (1–1.3)	0.016
MELD score	8 (6–12)	8 (6–12)	0.123
Child-Pugh score	5 (5–6)	5 (5–6)	0.440
Viral load (IU/ml x 10^6^)			
• Baseline	1.8 (0.0011–24	2.9 (0.013–3)	0.363
• Week 4 (lead in in TT)	0.002 (0–3.8)	0 (0–0.0008)	0.000
• Week 8	0 (0–0.013	0	0.851
Response to treatment; n (%)			0.066
• SVR12	18 (64%	11 (92%)	
• Relapse	3 (11%)	1 (8%)	
• Breakthrough	4 (14%)		
• Stopping rule	1 (4%)		
• Stop due to adverse events	2 (7%)		
Side effects; n (%)			0.000
• Anemia	8 (35%)	0	0.021
• Neutropenia	2 (9%)	0	0.425
• Thrombocytopenia	4 (17%)	0	0.169
• Infections	3 (13%)	0	0.271

Continuous data are expressed as median (range). PegIFN: Pegylated interferon, RBV: Ribavirin, TVR: Telaprevir, BOC: Boceprevir, SMV: Simeprevir, SOF: Sofosbuvir. AST: Aspartate-amino transferase, ALT: Alanin-amino transferase, INR: International Normalized Ratio, MELD: Model for End-Stage Liver disease, TT: Triple Therapy

The study was approved by the Ethical Committee of the Hospital Clínic Barcelona and all the participants provided written informed consent.

### Antiviral therapy

First wave, first generation, PIs (TVR and BOC) were administered with PegIFN and RBV as triple therapy (TT) until August 2014, when SMV, a second wave first generation PI became available. The antiviral treatment with TT was indicated in patients with HCV-related chronic liver disease (fibrosis stage ≥2) or compensated cirrhosis. The contraindications to TT in cirrhotic patients were: the presence of clinically significant portal hypertension as determined by an HVPG ≥10 mmHg, the clinical evidence of portal hypertension (ascites, varices), a platelet count <90,000 mm^3^, and a reduced synthetic liver function (assessed by bilirubin levels >2 mg/dL or albumin levels <35 g/L). Starting from November 2014, IFN-free regimens were available and patients with cirrhosis were prioritized to receive IFN-free therapy with SOF plus SMV with or without RBV for 12 or 24 weeks. None of the patients were on antibiotic prophylaxis.

The first group of patients enrolled in the study (Group A) received TT with PegIFN α2a (180 μg/week) and RBV (1000 or 1200 mg/d according to body weight) plus either TVR (n = 10; 1125 mg bid), BOC (n = 2; 800 mg tid), or SMV (n = 16; 150 mg qd). In order to dissect the potential effect of the PI during antiviral therapy, all patients received a 4 week lead-in period of antiviral therapy with PegIFN and RBV before the addition of the PI. The duration of TT was decided according to the specific package insert recommendations. The second group of patients (n = 12; Group B) received an IFN-free regimen with SOF 400 mg daily plus SMV 150 mg daily, with or without RBV (1000 mg or 1200 mg daily according to body weight) for 12 or 24 weeks.

### Monitoring and blood samples

All patients had outpatient clinic visits at baseline (day of treatment start), every 4 weeks during therapy, and at weeks 4, 12 and 24 after the end of treatment. Clinical, and virological data as well as any adverse event of special interest or that required a prescription medication for management (cutaneous rash, anemia [hemoglobin <10 g/dL], infections) throughout treatment and up to 24 weeks after the end of treatment, were recorded. HCV-RNA was measured by quantitative polymerase chain reaction with a limit of quantification of 15 IU/mL (VERSANT HCV RNA 1.0 Assay, Siemmens). Sustained virological response (SVR) defined as HCV-RNA <15 IU/mL that persisted 12 weeks after the end of antiviral treatment.

Blood samples (15 mL) from patients who underwent TT were prospectively obtained at baseline, week 4 (at the end of lead-in phase), week 8 (4 weeks after PI initiation), and week 12 of therapy (in 8 patients receiving TT with SMV). Blood samples from patients who received IFN-free treatment, were prospectively collected at baseline and week 4 of treatment.

### Phagocytic and oxidative burst activities of neutrophils and monocytes

In order to determine the phagocytosis of neutrophils and monocytes, Phagotest^®^ kit was used. The kit contains fluorescein-labeled opsonized *E*. *coli* bacteria and other reagents, as reported in the manufacturer’s description. Briefly, heparinized whole blood, processed within 2 h of sampling, was incubated with the FITC-labeled E. coli bacteria at 37°C and a negative control sample remained on ice. The phagocytosis was stopped by placing the samples on ice and adding quenching solution. This solution allowed the discrimination between attachment and internalization of bacteria by quenching the FITC fluorescence of surface bound bacteria leaving the fluorescence of internalized particles unaltered. After two washing steps with washing solution erythrocytes were then removed by addition of lysing solution. Data were analyzed by flow cytometry (BD FACS Canto^™^) evaluating 2 parameters as recommended in the kit: 1) the proportion of cells undergoing phagocytosis in general (phagocytosis rate, P-R), expressed as percentage and indicating the ingestion of one or more bacteria per cell, and 2) the individual phagocytic activity per cell (the number of bacteria engulfed per cell), corresponding to the median fluorescence intensity (P-MFI). The number of cells recorded per experiment ranged between 15.000 and 20.000. An example of the gating strategy is shown in S1 Fig.

In order to measure the oxidative burst activity of neutrophils and monocytes, Phagoburst kit^®^ was employed. The kit contains unlabeled opsonized *E*. *coli* bacteria as particulate stimulus, the protein kinase C ligand Phorbol 12-Myristate 13-Acetate (PMA) as high stimulus, the chemotactic peptide N-formyl-MetLeuPhe (fMLP) as low physiological stimulus, dihydrorhodamine (DHR)-123 as a fluorogenic substrate and other reagents as reported in the manufacturer’s description. Briefly, heparinized whole blood, processed within 2 h of sampling, was incubated with the various stimuli at 37°C, a sample without stimulus served as negative control. Upon stimulation with unlabeled *E*.*coli*, neutrophils and monocytes produced reactive oxygen metabolites (superoxide anion, hydrogen peroxide, hypochlorous acid) which destroyed bacteria inside the phagosome. Formation of the reactive oxidants during the oxidative burst has been monitored by the addition and oxidation of DHR-123. The reaction was stopped by addition of lysing solution, which removed erythrocytes and allowed a partial fixation of cells. Data were analyzed by flow cytometry (BD FACS Canto^™^) measuring 2 parameters: 1) the proportion of cells having produced reactive oxygen radicals (burst rate, B-R), expressed as percentage, and 2) the burst enzymatic activity per cell, corresponding to the median fluorescence intensity (B-MFI). The number of cells recorded per experiment ranged between 15.000 and 20.000.

### In vitro studies of phagocytic and oxidative burst activities of neutrophils and monocytes

To evaluate the isolated effect of IFN and PIs on phagocytic and oxidative burst capacity of neutrophils and monocytes, heparinized whole blood from four cirrhotic patients and four healthy controls was incubated at 37°C during 3h with or without (negative control): SMV (5μM and 0.05μM, OLYSIO^®^ Janssen); TVR (5μM and 0.05μM, INCIVO^®^ Janssen) and IFN-αA/D (50U/mL and 5000U/mL, Sigma-Aldrich). Drug stock solutions were prepared in DMSO and working dilutions were done in DMEM. Phagotest^®^ and Phagoburst^®^ were performed as reported above. Each experiment was performed in duplicate.

### Measurement of cytokine secretion by neutrophils

#### Cells isolation

Neutrophils were isolated from the whole blood into a band free from red blood cells, using Polymorphprep^™^ solution. Briefly, a layer 5.0 ml of anticoagulated whole blood were layered over 5.0 ml of Polymorphprep^™^ in a 15 ml centrifuge tube and centrifuged at 1000 x g for 45 minutes in a swing-out rotor at 18–22°C. After centrifugation, two leucocyte bands were visible: the top band of peripheral blood mononuclear cells and the lower band of neutrophils, while the erythrocytes were pelleted. The neutrophil band was harvested using a Pasteur pipette and diluted by addition of culture medium in a 15 ml tube. The cells were spun down twice (at 18–22°C, 400 x g, 10 min) and then re-suspended in medium.

#### Toll-like receptor-5 stimulation

After cell isolation and count to obtain 10^6^ cells/mL, 100 μL of the cells were added to 96-well culture plates and were stimulated with 1 μL of flagellin (concentration 100μg/mL, FLA), a monomeric constituent of bacterial flagella, that is a TLR-5 agonist. The cells, with and without FLA, were incubated at 37°C for 8 hours.

#### Cytokine determination

The supernatant of neutrophils was collected separately and cryopreserved to -80° for further analysis. Quantification of specific cytokines secretion (IL-1b, IL-6, IL-10, IL-12) was performed using Luminex^®^. The supernatant of unstimulated neutrophils was used as control for Luminex^®^ determinations.

### Statistical analysis

Continuous variables are depicted using median and ranges (or mean with standard deviation), and categorical variables are expressed as absolute numbers and percentages. Mann-Whitney non-parametric test was used to compare data from healthy controls and baseline data from the patients included in the study. A one-way repeated measures ANOVA was used to determine whether there was a statistical difference in P-R, P-MFI, B-R, B-MFI, IL-1b, IL-6, IL-10, and IL-12 over the course of the 3 time points during antiviral therapy (baseline, W4, W8) in patients treated with IFN (Group A). Post hoc tests with Bonferroni adjustments were used to examine the pairwise combinations. When the assumption of normality was violated, a logarithmic transformation of the variable was performed. If despite the transformation, the variable continues having a no-normal distribution comparisons between groups were performed using the Friedman test (P-R and B-R in neutrophils, and P-MFI and B-MFI in monocytes). Again pairwise comparisons were performed with a Bonferroni correction for multiple comparisons. The Wilcoxon test was used to analyze the difference between the 2 time points in Group B patients. All differences were considered significant at p-value of <0.05. Statistical analyses were performed with SPSS, version 20 (SPSS, Chicago, IL).

## Results

### Effectiveness and tolerability of antiviral therapy

Sixty-four percent and 92% of the patients included in Groups A and B, respectively, achieved SVR. During antiviral treatment, anemia (n = 8), neutropenia (n = 2), thrombocytopenia (n = 4), and 3 episodes of bacterial infections of minor clinical relevance (urinary [n = 1], gastrointestinal [n = 1] and skin [n = 1]) with no positive cultures were observed only in Group A patients. No hematological or clinical adverse events were observed in Group B patients.

### Phagocytic activity of neutrophils and monocytes

At baseline, patients in Group A (n = 28) had significantly lower rates of neutrophils undergoing phagocytosis (P-R) as compared to healthy controls (n = 10) (p = 0.006). However, there were no differences in the number of bacteria engulfed per cell (P-MFI) between patients and healthy controls at baseline. Neutrophil P-R and P-MFI did not change throughout the study period in both groups of patients (S2A–S2D Fig). Likewise, monocyte P-R was similar between controls and patients and it did not change in the specific time-points in both groups (S2E and S2F Fig). In contrast, monocyte P-MFI was significantly higher at baseline as compared to controls in Group B patients (p = 0.043, S2H Fig). No changes were observed during the study period in both groups of patients (S2E–S2H Fig). Raw data and *p* values for pairwise comparisons are shown in S1 and S2 Tables. In patients from Group A, there were no significant changes in both parameters according the type of PI administered (data not shown).

### Oxidative burst activity of neutrophils and monocytes

**Fig 1:**
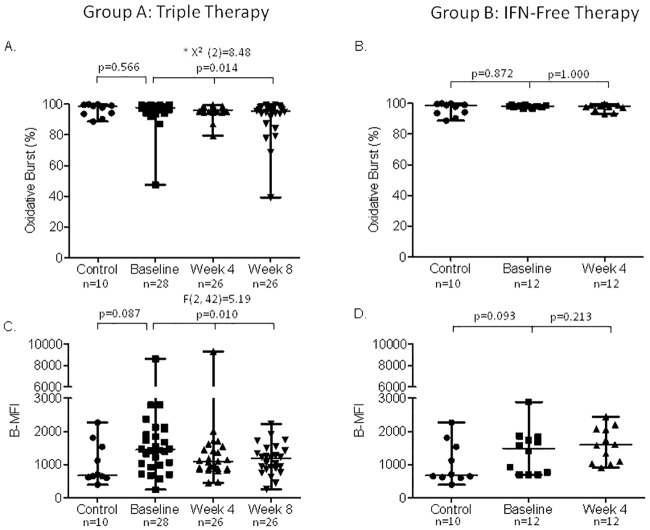
Oxidative burst capacity of neutrophils. Panel A and B show the burst rate (B-R) in patients treated with triple therapy and IFN-free regimen, respectively. Panel C and D show the enzymatic activity per cell (median fluorescence intensity, B-MFI) in patients treated with triple therapy and IFN-free regimen, respectively. Data are analyzed at baseline (before starting antiviral therapy), and at week 4 and 8 of therapy. * These comparisons were performed by Friedman tests.

**Fig 2:**
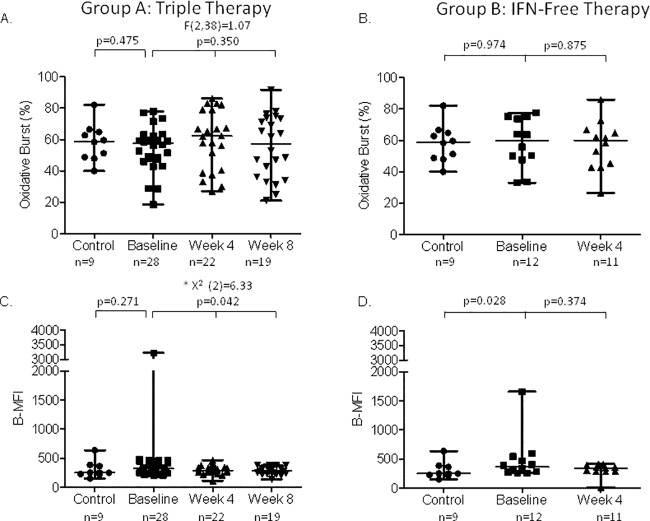
Oxidative burst capacity of monocytes. Panel A and B show the burst rate (B-R) in patients treated with triple therapy and IFN-free regimen, respectively. Panel C and D show the enzymatic activity per cell (median fluorescence intensity, B-MFI) in patients treated with triple therapy and IFN-free regimen, respectively. Data are analyzed at baseline (before starting antiviral therapy, n = 28), and at week 4 and 8 of therapy. * These comparisons were performed by Friedman tests.

At baseline, there were no significant differences between patients and healthy controls in both groups (Fig 1A–1D). In Group A, neutrophil burst rate (B-R) significantly decreased throughout the study period (p = 0.014) (Fig 1A). Pairwise comparisons evidenced a decrease between baseline and week 4 of therapy at the limit of significance (p = 0.063) and a statistically significant decrease between baseline and week 8 of therapy (p = 0.023). No differences were observed between week 4 and 8 of therapy indicating no effect of the PI introduction. Similarly, oxidative enzymatic activity per cell (B-MFI) significantly decreased during the study period (p = 0.010) with a decrease at week 4 and 8 of therapy as compared to baseline (p = 0.056 and p = 0.018, respectively) (Fig 1C). There were no significant differences after the introduction of the PI (p = 1.000). In the 8 patients with blood sample at week 12 of therapy (TT with SMV), there was also a significant decline of B-MFI as compared to baseline (p = 0.028) but not different from the results at week 4 or 8 of therapy (data not shown). Monocyte B-R did not change in Group A patients (Fig 2A), while monocyte B-MFI decreased during the study period (p = 0.042) (Fig 2C). Pairwise comparisons revealed a significant decrease at week 8 of therapy as compared to baseline (p = 0.037). On the other hand, in Group B patients, neutrophil and monocyte B-R and B-MFI did not change throughout the study period (Figs 1B, 1D, 2B and 2D). Raw data and *p* values for pairwise comparisons are shown in S1 and S2 Tables.

### In vitro experiments

As shown in S3A–S3D Fig P-R, B-R and B-MFI in neutrophils were similar in blood from cirrhotic patients and healthy controls. However, P-MFI was lower in cirrhotic patients as compared to healthy controls. In both groups of patients, there were no differences in P-R, B-R, P-MFI and B-MFI of neutrophils after incubation of human blood with 2 different concentrations of interferon, TVR and SMV as compared to blood incubated with DMEM. In monocytes, there were no differences in P-R, B-R, P-MFI and B-MFI between cirrhotic patients and healthy controls (S4A–S4D Fig). The percentage of monocytes engulfing bacteria (P-R) decreased with the addition of 50U and 5000U of IFN as compared to that of monocytes from blood incubated only with DMEM (33% vs. 54% and 44% vs. 54%, respectively) (S4A Fig). In addition, there was a decrement in the percentage of monocytes producing reactive oxygen radicals (B-R) with the addition of IFN as compared to that of monocytes from blood incubated only with DMEM (30% vs. 51% and 42% vs. 51%, respectively) (S4C Fig). None of these changes were observed with the addition of different concentrations of a PI (TVR or SMV). Regarding P-MFI and B-MFI in monocytes, there were no significant changes after the addition of IFN or a PI (S4B and S4D Fig).

### Cytokine secretion

Regarding innate immune recognition, we assessed the secretion of IL-10, IL-12, IL-1b, IL-6, TNFα by neutrophils before and after stimulation with FLA. The secretion of IL-10 and IL-12 remained at the lower limit of detection even after stimulation; therefore they were not considered in the final analysis. In both groups, neutrophils stimulation with FLA did not induce a significant change in IL-6, TNFα and IL-1b secretion throughout the study time-points (neither after 4 weeks of lead-in with PegIFN and RBV nor after the introduction of the PI) (data not shown).

## Discussion

Real-life data have strongly suggested that an increased number of bacterial infections occurs in patients with cirrhosis receiving triple antiviral therapy with TVR or BOC, especially those with low platelets and low albumin levels [7–9]. Indeed, in a previous study we found that TT was the only independent predictor of bacterial infections in a group of cirrhotic patients receiving this antiviral regimen (25% vs. 9% in patients treated with dual therapy with PegIFN and RBV; p = 0.001). Interestingly, there was also a change in the pattern of infections with an increase in the number of respiratory tract infections (12% vs. 1%; p = 0.049) which is different from the infections commonly observed in patients with cirrhosis (whether or not receiving antiviral therapy with an IFN-based regimen) [9, 16, 17].

In this prospective study we hypothesized that neutrophil and monocyte dysfunction induced by a deleterious effect of anti-HCV PIs, could partly explain the increase in bacterial infections observed in patients treated with a PI-containing regimen. Our hypothesis was supported by the fact that anti-HIV PIs have been shown to impair neutrophil functions including phagocytosis, superoxide production and chemotaxis, and neutrophil apoptosis *in vitro* [14]. Moreover, a recently published study evaluated the neutrophil function in patients receiving triple antiviral therapy in comparison to patients with chronic hepatitis C treated with dual therapy with PegIFN and RBV [15]. As previously described, the authors observed a significantly higher number of infections in patients receiving TT (31%) as compared to patients receiving dual therapy (26%; p = 0.045). Interestingly, and from a pathogenic point of view, the authors also demonstrated a 40% decrease in neutrophil phagocytic capacity at the end of therapy in PI-treated patients that was not evidenced in patients treated with dual therapy. Phagocytic capacity recovered 24 weeks after treatment discontinuation. Oxidative burst was also lower in patients treated with TT (especially in those receiving TVR) as compared to the control group of patients on dual therapy.

Our results did not confirm the data by Spindelboeck et al as we did not find a significant decrease in neutrophil or monocyte phagocytic capacity during TT with TVR, BOC or SMV. Conversely, we observed a decrease in the proportion of cells producing reactive oxygen radicals (B-R) and in neutrophil oxidative enzymatic activity per cell (B-MFI) after 4 weeks of lead-in therapy with PegIFN and RBV as compared to baseline in Group A patients (treated with TT). This effect was more pronounced at week 8 but did not change significantly between weeks 4 and 8 of therapy (suggesting no effect of the addition of a PI). In monocytes, there was also a decrease in the enzymatic activity per cell (B-MFI) at week 8 of therapy as compared to baseline with no changes between week 4 and 8 (addition of the PI). None of these changes were observed in Group B patients (treated with an IFN-free regimen containing a PI). *In vitro* experiments conducted to confirm the clinical results, showed a decrease in P-R and B-R in monocytes (but not in neutrophils) with the addition of IFN and no changes with the addition of different concentrations of PIs.

Thus, our results suggest a predominant effect of IFN, rather than the PI, on neutrophils’ and monocytes’ function. IFN-based therapy has been clearly associated with an increased risk of infections, especially in patients with decompensated cirrhosis [16]. As described in detail previously, the explanation for this phenomenon is unknown [18–20]. Some studies including patients with chronic hepatitis C (with and without cirrhosis) have observed an increase in neutrophil oxidative burst and phagocytic capacity during therapy as compared to baseline values [21–23]. Giorgini et al, suggested that the enhancement in neutrophil and monocyte function during antiviral therapy with an IFN-based therapy could be explained by a compensatory effect to the IFN-induced bone marrow toxicity. In this sense, IFN therapy would select a more resistant population of neutrophils and monocytes with improved chemotactic and oxidative burst capacity [21]. Similarly, Jablonowska et al [22] and Piazzola et al [23] found an increase in the production of reactive oxygen species by neutrophils in patients treated with IFN. Moreover, several studies have observed monocyte activation, either *in vitro* after stimulation with IFN [24] or through the determination of biomarkers of monocyte activation (soluble CD14 or IL-18 levels) [25]. However, we found opposite results with a decline in the oxidative burst capacity of neutrophils and monocytes after IFN therapy. One explanation would be that in patients with cirrhosis, phagocytic cells are not able to activate such compensatory mechanisms. Cirrhotic patients present a state of immune paralysis secondary to the chronic stimulation of immune cells by microbial and damage-associated molecular patterns (MAMPs or DAMPs) leading to innate and adaptive immune cell exhaustion [11].

The *in vitro* experiments provided two major results: 1) The phagocytic activity per cell (P-MFI) was lower in blood from cirrhotic patients as compared to healthy controls. This result was not unexpected due to the well known impairment of phagocytosis observed in patients with advanced liver disease [11], and 2) There was a significant decline in B-R and P-R in monocytes (but not in neutrophils) after the incubation of blood from cirrhotic patients with 2 different concentrations of IFN that were not observed after the addition of a PI, which confirm the *in vivo* results. Surprisingly, these changes were not observed in neutrophils. The explanation for this phenomenon is unknown, but it is possible that in the experiment conditions, neutrophils were less sensitive to IFN as compared to monocytes. This has also been reported in a previous study in which the intensity of STAT1 fluorescence was lower in neutrophils and lymphocytes as compared to monocytes after stimulation with IFNγ and IFNα [26].

The differences in our results with the study by Spindelboeck et al [15] could also be explained by the differences in the design of both studies: 1) In contrast to theirs, our current study included predominantly patients with cirrhosis which could have some degree of baseline immune dysfunction, 2) Our study design was different, since we did not compare a cohort with TT vs. a cohort of patients receiving dual therapy with PegIFN and RBV, but all patients in our study population received a 4 week lead-in period of PegIFN and RBV making each patient its own control (this might avoid the potential inter-individual variations in immune responses), and 3) The current study has a limited number of data at week 12 of therapy in Group A patients (n = 8) which hampers the evaluation of a more prolonged use of PIs on immune function. On the other hand, the current results do not confirm the data from the real-life cohorts pointing PI-containing regimens as a risk factor for the development of bacterial infections. The possible explanation for these results is that despite our current study enrolled predominately patients with cirrhosis, the degree of liver dysfunction or portal hypertension in these patients is much lower than in those analyzed in previous cohorts [7, 9]. Indeed, only 1 and 12 patients the current study had albumin levels <35g/L and platelet counts <100,000, respectively. This would point towards an important role of liver disease severity in the pathogenesis of infections when using a PI-containing antiviral regimen. Moreover, this might also explain the relative low magnitude of the changes in neutrophil and monocyte oxidative enzymatic activity observed during antiviral therapy in the current study. It is possible that the analysis of neutrophil and monocyte function in patients with more advanced liver disease would have shown a more pronounced impairment in phagocytosis or oxidative burst capacity during antiviral therapy. Unfortunately, this is difficult to demonstrate because the use IFN is contraindicated in patients with liver dysfunction.

The main limitations of our manuscript are: 1) IFN-based therapies in combination with a PI are not longer administered in most of the countries worldwide, 2) The small sample size of the current cohort that was related to the rapid changes in the antiviral regimen prescribed during the study period, 3) The small number of patients with bacterial infections observed during the course of therapy in patients included in this study which hampers the comparisons of phagocytic and oxidative burst capacity between patients with and without infections, and 4) We did not analyze other mechanisms involved in the immune pathogenesis of bacterial infections such as chemotaxis or migration of immune cells to the site of infection, or a potential deleterious effect of PI on other cells such as NK, γδ T cells or dendritic cells. Thus, we cannot completely rule-out the negative effect of PI in the immune system.

Despite these results, assessing the potential causes of an increased number of infections in patients receiving a PI is still relevant for several reasons. First, infections have also been reported in patients receiving a PI as part of an IFN-free regimen (SMV plus SOF) [10]. Secondly, PIs still are an important component of current and future IFN-free regimens (i.e. SMV in combination with SOF, Paritaprevir/r in combination with Ombitasvir with or without Dasabuvir, Grazoprevir plus Elbasvir, etc).

In conclusion, our data suggest that the use IFN (rather than the PI) has a deleterious effect on neutrophil and monocyte phagocytic and oxidative burst capacity in patients with hepatitis C-related advanced liver fibrosis receiving antiviral therapy.

## Supporting Information

S1 Fig Gating strategy. (PDF)

S2 Fig Phagocytic capacity of neutrophils (Panels A-D) and monocytes (Panels E-H). Panel A, B, E and F show the rate of phagocytic capacity (P-R) in patients treated with triple therapy and IFN-free regimen. Panel C, D, G and H show the number of bacteria engulfed by cell (median fluorescence intensity, P-MFI) in patients treated with triple therapy and IFN-free regimen. Data are analyzed at baseline (before starting antiviral therapy), and at week 4 (treatment only with PegIFN and RBV) and 8 (TT) of therapy. * These comparisons were performed by Friedman tests.
(PPTX)

S3 Fig In vitro data of phagocytic and oxidative burst capacity in neutrophils with different concentrations of interferon, simeprevir and telaprevir. Panel A shows the rate of phagocytic capacity (P-R). Panel B shows the number of bacteria engulfed by cell (median fluorescence intensity, P-MFI). Panel C shows the burst rate (B-R). Panel D shows the enzymatic activity per cell (median fluorescence intensity, B-MFI). Black bars expressed the results in blood from cirrhotic patients and grey bars expressed the results in blood from healthy controls. Data are expressed as mean and standard deviation. IFN50: Interferon 50 U/mL, IFN5000: Interferon 5000 U/mL, SMV0.05: Simeprevir 0.05 μM, SMV5: Simeprevir 5 μM, TVR0.05: Telaprevir 0.05 μM, TVR5: Telaprevir 5 μM.
(PPTX)

S4 Fig In vitro data of phagocytic and oxidative burst capacity in monocytes with different concentrations of interferon, simeprevir and telaprevir. Panel A shows the rate of phagocytic capacity (P-R). Panel B shows the number of bacteria engulfed by cell (median fluorescence intensity, P-MFI). Panel C shows the burst rate (B-R). Panel D shows the enzymatic activity per cell (median fluorescence intensity, B-MFI). Black bars expressed the results in blood from cirrhotic patients and grey bars expressed the results in blood from healthy controls. Data are expressed as mean and standard deviation. IFN50: Interferon 50 U/mL, IFN5000: Interferon 5000 U/mL, SMV0.05: Simeprevir 0.05 μM, SMV5: Simeprevir 5 μM, TVR0.05: Telaprevir 0.05 μM, TVR5: Telaprevir 5 μM.
(PPTX)

S1 Table Raw data of phagocytosis and oxidative burst capacity. (DOCX)

S2 Table Individual data on phagocytic and oxidative burst capacity. (DOCX)
