# Correction: Neutrophil and Monocyte Function in Patients with Chronic Hepatitis C Undergoing Antiviral Therapy with Regimens Containing Protease Inhibitors with and without Interferon

**DOI:** 10.1371/journal.pone.0170913

**Published:** 2017-01-20

**Authors:** Martina Gambato, Noelia Caro-Pérez, Patricia González, Nuria Cañete, Zoe Mariño, Sabela Lens, Martín Bonacci, Concepció Bartres, José-María Sánchez-Tapias, José A. Carrión, Xavier Forns, Manel Juan, Sofía Pérez-del-Pulgar, María-Carlota Londoño

There are errors in the Funding section. The correct funding information is as follows: SPP has received a grant from Instituto de Salud Carlos III, Ministerio de Economía y Competitividad (PI13/00155), co-funded by Fondo Europeo de Desarrollo Regional (FEDER), Unión Europea, Una manera de hacer Europa. XF has received a grant from L' Agència de Gestió d’Ajuts Universitaris i de Recerca (AGAUR) (2014-SGR-605). XF was also supported in part by grant PI15/00151, integrated in Plan Nacional de I+D+I and funded by ISCIII-Subdirección General de Evaluación y el Fondo Europeo de Desarrollo Regional (FEDER-"Una manera de Hacer Europa"). MG was funded with an EASL Entry Level Research Fellowship (Dame Sheila Sherlock EASL Fellowship Programme).
