# Supplementary figures and images for: Neutrophil and Monocyte Function in Patients with Chronic Hepatitis C Undergoing Antiviral Therapy with Regimens Containing Protease Inhibitors with and without Interferon
Source: PLoS One. 2016 Nov 18;11(11):e0166631. doi: 10.1371/journal.pone.0166631 (PMC5115763; doi:10.1371/journal.pone.0166631)

Supplementary Figure 1

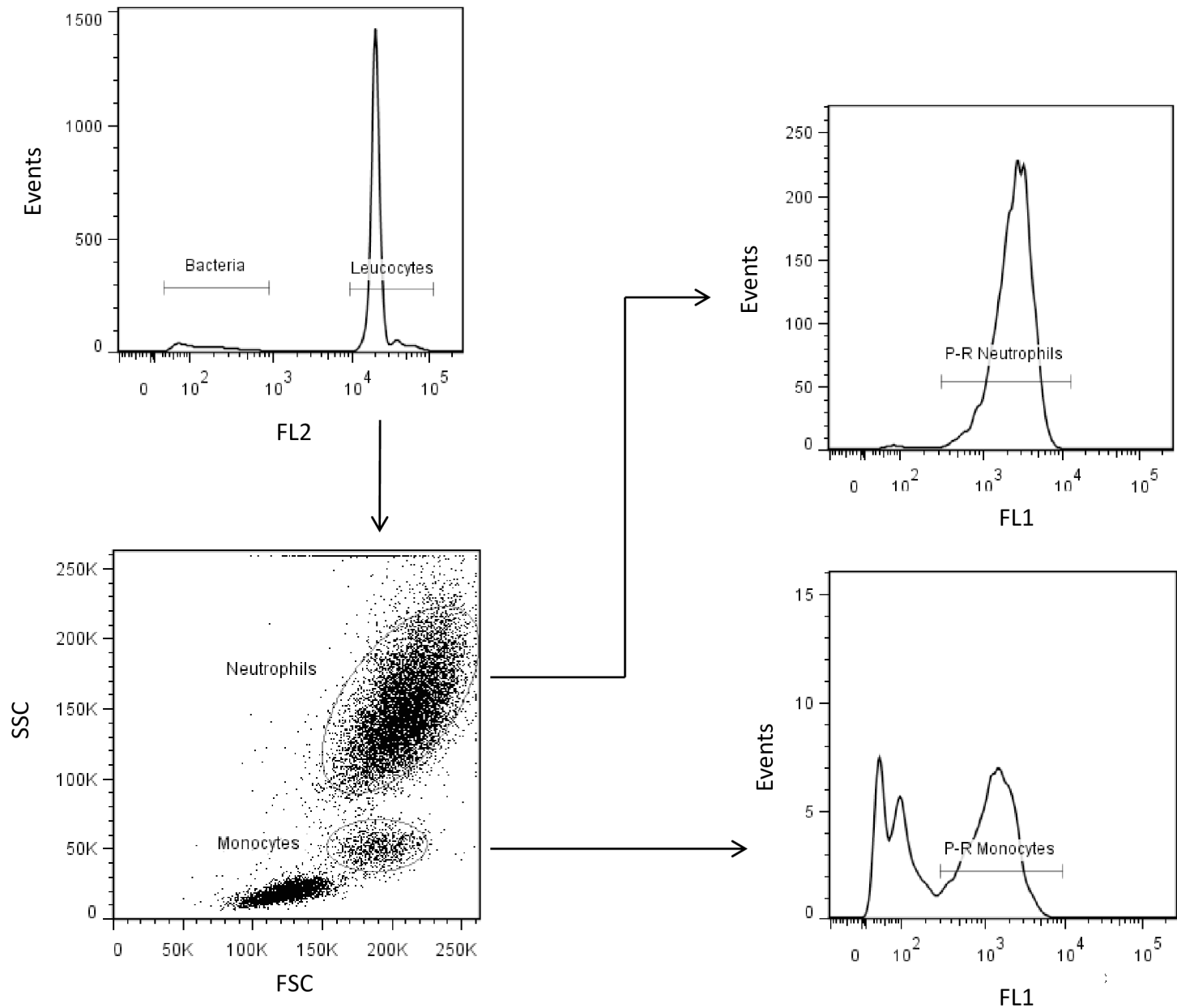

Supplement: S1 Fig — (PDF) [file pone.0166631.s001.pdf]

## Slide 1
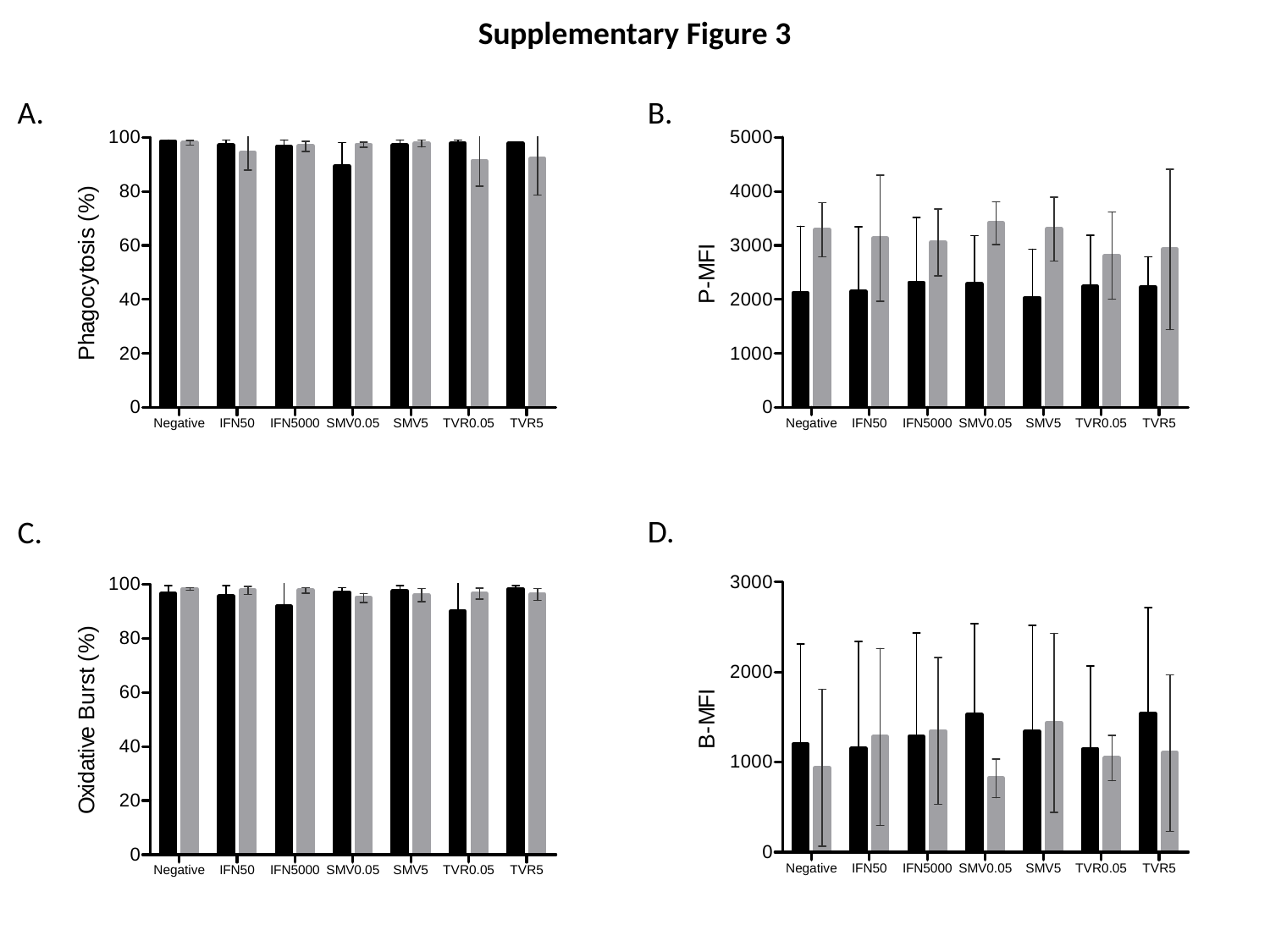

Supplementary Figure 3
A.
B.
D.
C.

Supplement: S3 Fig — Panel A shows the rate of phagocytic capacity (P-R). Panel B shows the number of bacteria engulfed by cell (median fluorescence intensity, P-MFI). Panel C shows the burst rate (B-R). Panel D shows the enzymatic activity per cell (median fluorescence intensity, B-MFI). Black bars expressed the results in blood from cirrhotic patients and grey bars expressed the results in blood from healthy controls. Data are expressed as mean and standard deviation. IFN50: Interferon 50 U/mL, IFN5000: Interferon 5000 U/mL, SMV0.05: Simeprevir 0.05 μM, SMV5: Simeprevir 5 μM, TVR0.05: Telaprevir 0.05 μM, TVR5: Telaprevir 5 μM. (PPTX) [file pone.0166631.s003.pptx]

## Slide 1
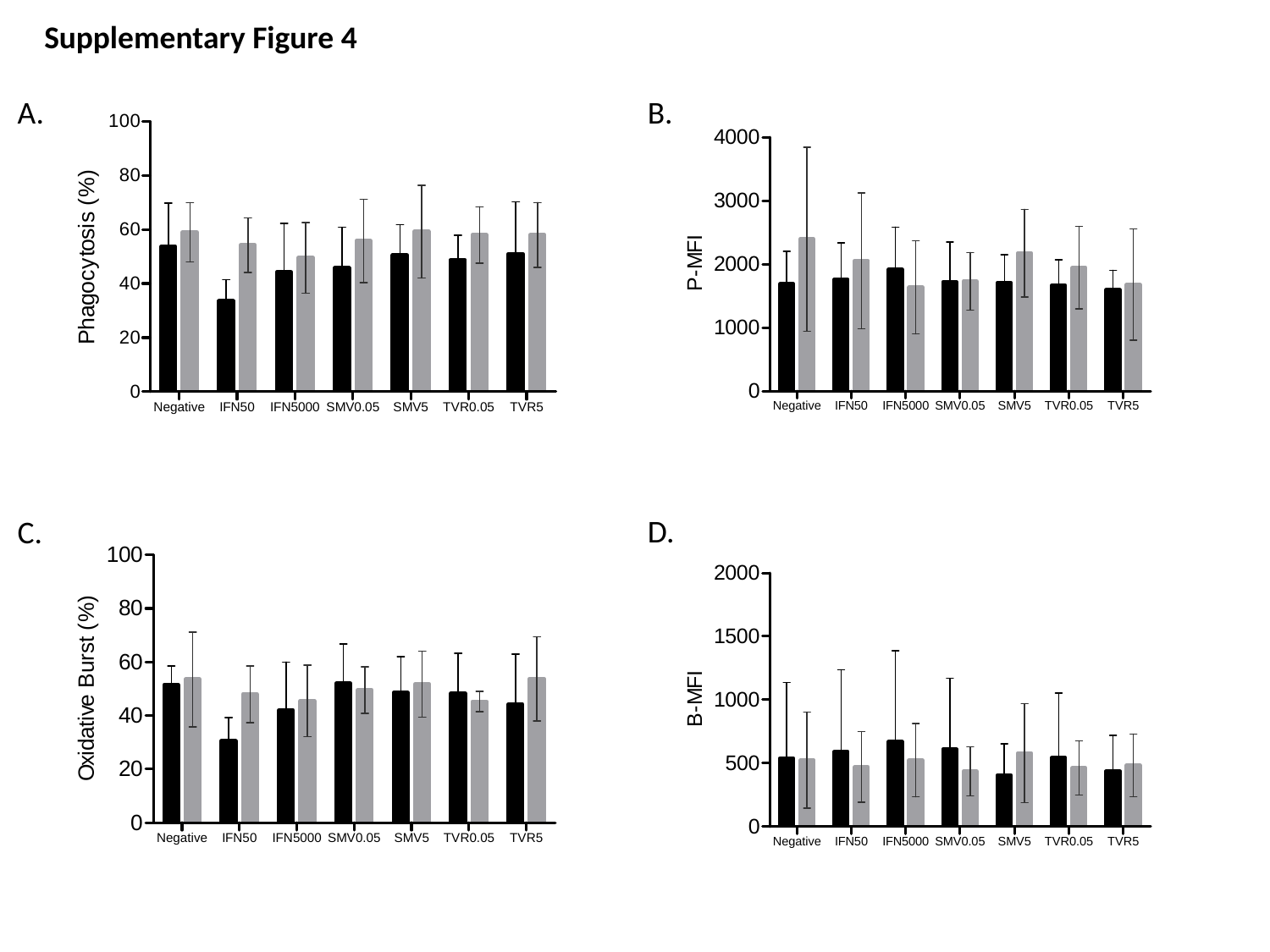

Supplementary Figure 4
A.
B.
D.
C.

Supplement: S4 Fig — Panel A shows the rate of phagocytic capacity (P-R). Panel B shows the number of bacteria engulfed by cell (median fluorescence intensity, P-MFI). Panel C shows the burst rate (B-R). Panel D shows the enzymatic activity per cell (median fluorescence intensity, B-MFI). Black bars expressed the results in blood from cirrhotic patients and grey bars expressed the results in blood from healthy controls. Data are expressed as mean and standard deviation. IFN50: Interferon 50 U/mL, IFN5000: Interferon 5000 U/mL, SMV0.05: Simeprevir 0.05 μM, SMV5: Simeprevir 5 μM, TVR0.05: Telaprevir 0.05 μM, TVR5: Telaprevir 5 μM. (PPTX) [file pone.0166631.s004.pptx]
