# Supplementary material for: Neutrophil and Monocyte Function in Patients with Chronic Hepatitis C Undergoing Antiviral Therapy with Regimens Containing Protease Inhibitors with and without Interferon
Source: PLoS One. 2016 Nov 18;11(11):e0166631. doi: 10.1371/journal.pone.0166631 (PMC5115763; doi:10.1371/journal.pone.0166631)
Supplement: S2 Fig — Panel A, B, E and F show the rate of phagocytic capacity (P-R) in patients treated with triple therapy and IFN-free regimen. Panel C, D, G and H show the number of bacteria engulfed by cell (median fluorescence intensity, P-MFI) in patients treated with triple therapy and IFN-free regimen. Data are analyzed at baseline (before starting antiviral therapy), and at week 4 (treatment only with PegIFN and RBV) and 8 (TT) of therapy. * These comparisons were performed by Friedman tests. (PPTX) [file pone.0166631.s002.pptx]

## Slide 1
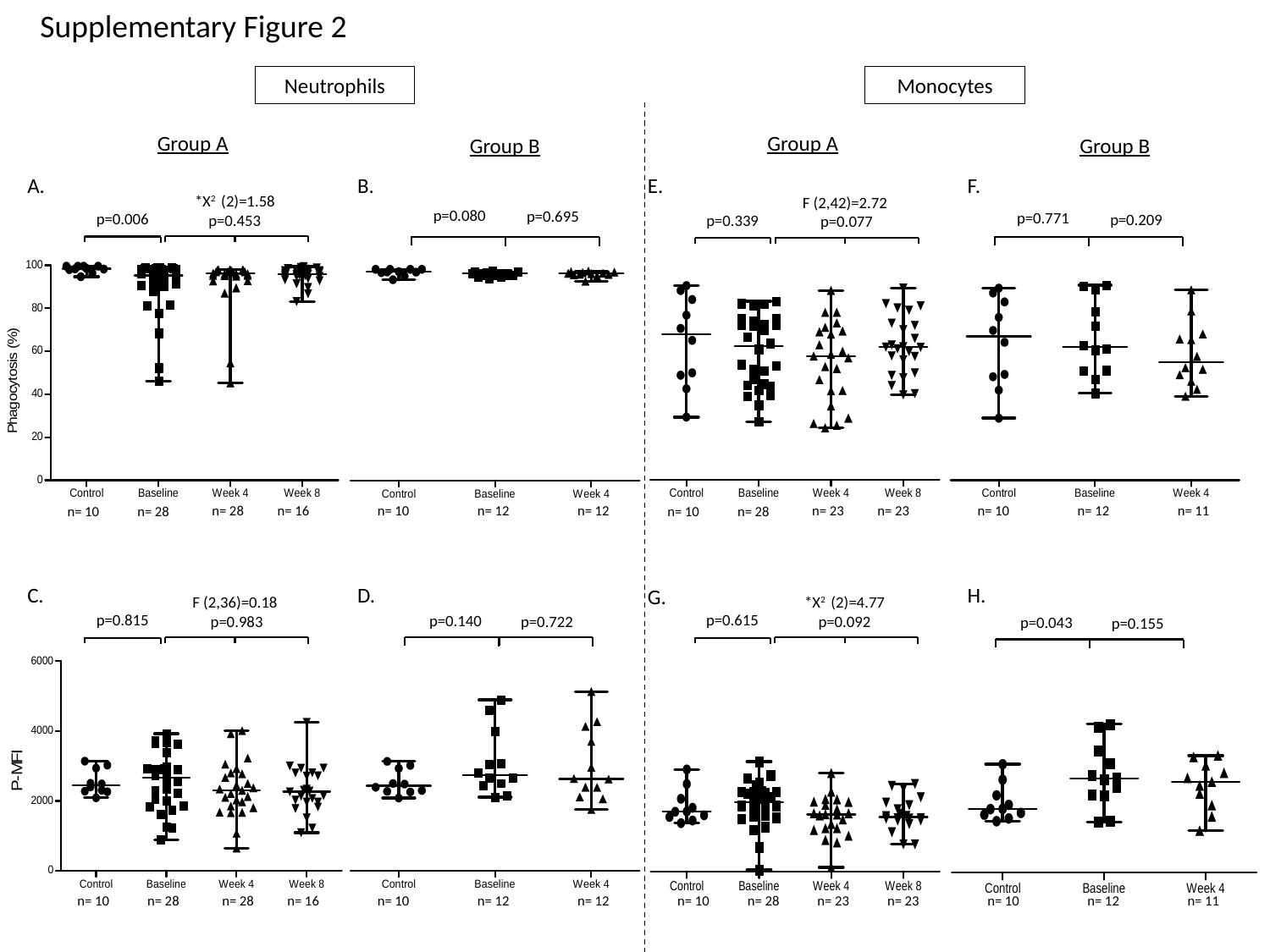

Supplementary Figure 2
Neutrophils
Monocytes
Group A
Group A
Group B
Group B
A.
B.
E.
F.
*X2 (2)=1.58
p=0.453
F (2,42)=2.72
 p=0.077
p=0.080
p=0.695
p=0.771
p=0.006
p=0.209
p=0.339
n= 28
n= 16
n= 10
n= 12
n= 12
n= 23
n= 23
n= 10
n= 12
n= 11
n= 10
n= 28
n= 10
n= 28
C.
D.
H.
G.
F (2,36)=0.18
 p=0.983
*X2 (2)=4.77
p=0.092
p=0.815
p=0.615
p=0.140
p=0.722
p=0.043
p=0.155
n= 28
n= 16
n= 23
n= 23
n= 10
n= 28
n= 10
n= 12
n= 12
n= 10
n= 28
n= 10
n= 12
n= 11
