# Supplementary material for: Neutrophil and Monocyte Function in Patients with Chronic Hepatitis C Undergoing Antiviral Therapy with Regimens Containing Protease Inhibitors with and without Interferon
Source: PLoS One. 2016 Nov 18;11(11):e0166631. doi: 10.1371/journal.pone.0166631 (PMC5115763; doi:10.1371/journal.pone.0166631)
Supplement: S1 Table — (DOCX) [file pone.0166631.s005.docx]

**Supplementary Table 1.** Raw data of phagocytosis and oxidative burst capacity

| **Variable** | **Healthy Controls** | **Group A**  **Baseline Week 4 Week 8**   1. **(2) (3)** | | | **p value**  **(1vs.2)** | **p value**  **(1vs.3)** | **p value**  **(2vs.3)** | **Group B**  **Baseline Week 4**   1. **(2)** | | **p value**  **(1vs.2)** |
| --- | --- | --- | --- | --- | --- | --- | --- | --- | --- | --- |
| **Neutrophils** |  | | | | | | | | | |
| **P-R*^¥^** | 98 (95-99) | 95 (46-99) | 96 (45-99) | 96 (83-99) | - | - | - | 98 (95-98) | 98 (94-98) | 0.695 |
| **P-MFI** | 2448 (2091-3140) | 2572 (890-3927) | 2300 (650-4010) | 2267 (1097-4257) | 1.000 | 1.000 | 1.000 | 2750 (2120-4907) | 2637 (1762-5141) | 0.722 |
| **B-R*** | 98 (89-99) | 98 (47-99) | 96 (79-99) | 96 (39-99) | 0.063 | 0.023 | 1.000 | 98 (97-99) | 98 (93-99) | 1.000 |
| **B-MFI** | 680 (395-2269) | 1430 (250-8612) | 1094 (454-9325) | 1200 (261-2229) | 0.056 | 0.018 | 1.000 | 1492 (694-2890) | 1595 (904-2446) | 0.213 |
| **Monocytes** |  | | | | | | | | | |
| **P-R** | 67 (28-89) | 53 (27-82) | 57 (24-87) | 61 (39-88) | 0.505 | 1.000 | 0.112 | 62 (40-91) | 55 (39-88) | 0.209 |
| **P-MFI*^¥^** | 1499 (1212-2557) | 1682 (39-2750) | 1426 (95-2465) | 1361 (681-2195) | - | - | - | 2223 (1193-3506) | 2143 (989-2763) | 0.155 |
| **B-R** | 59 (40-82) | 56 (19-78) | 62 (27-86) | 57 (21-96) | 1.000 | 1.000 | 0.691 | 58 (33-78) | 60 (27-86) | 0.875 |
| **B-MFI*** | 252 (150-638) | 312 (203-3219) | 364 (257-1619) | 282 (137-380) | 0.952 | 0.037 | 0.401 | 364 (257-1659) | 345 (12-415) | 0.374 |

*These comparisons were analyzed by Friedman test. ^¥^ Pairwise comparisons were not performed because the overall test retained the null hypothesis of no differences.
