# Supplementary material for: Neutrophil and Monocyte Function in Patients with Chronic Hepatitis C Undergoing Antiviral Therapy with Regimens Containing Protease Inhibitors with and without Interferon
Source: PLoS One. 2016 Nov 18;11(11):e0166631. doi: 10.1371/journal.pone.0166631 (PMC5115763; doi:10.1371/journal.pone.0166631)
Supplement: S2 Table — (DOCX) [file pone.0166631.s006.docx]

**Supplementary Table 2.** Individual data on phagocytic and oxidative burst capacity

| **Neutrophils** | | | | | | | | | | | | | | | | | | | | | | | |
| --- | --- | --- | --- | --- | --- | --- | --- | --- | --- | --- | --- | --- | --- | --- | --- | --- | --- | --- | --- | --- | --- | --- | --- |
| **P-R** | | | | | | **P-MFI** | | | | | | **B-R** | | | | | | **B-MFI** | | | | | |
| HC | Group A | | | Group B | | HC | Group A | | | Group B | | HC | Group A | | | Group B | | HC | Group A | | | Group B | |
|  | W0 | W4 | W8 | W0 | W4 |  | W0 | W4 | W8 | W0 | W4 |  | W0 | W4 | W8 | W0 | W4 |  | W0 | W4 | W8 | W0 | W4 |
| 99,60 | 96,80 | 97,03 |  | 97,60 | 98,32 | 3140 | 4907 | 5141 |  | 3056 | 2404 | 99,00 | 86,95 | 87,00 | 84,30 | 98,65 | 99,00 | 1122 | 8612 | 1088 | 1746 | 1407 | 1093 |
| 99,60 | 97,80 | 97,28 |  | 97,70 | 97,68 | 3026 | 4623 | 4142 |  | 2120 | 2120 | 98,90 | 98,00 | 96,20 | 98,70 | 98,52 | 99,20 | 395 | 2800 | 900 | 890 | 1578 | 1578 |
| 99,60 | 96,10 | 96,52 |  | 98,50 | 98,82 | 2944 | 4010 | 1762 |  | 2173 | 2068 | 99,90 | 99,53 | 97,10 | 94,92 | 98,92 | 99,50 | 2269 | 1437 | 1175 | 762 | 694 | 1334 |
| 99,60 | 98,20 | 96,92 | 95,55 | 97,80 | 97,28 | 2505 | 3927 | 4010 | 2267 | 4623 | 4142 | 99,50 | 97,47 | 95,30 | 96,72 | 97,48 | 93,60 | 1814 | 1550 | 1150 | 943 | 1684 | 1612 |
| 98,50 | 98,60 | 96,30 | 91,48 | 97,40 | 93,93 | 2493 | 3740 | 4000 | 2140 | 3092 | 2971 | 99,80 | 47,40 |  | 97,85 | 96,73 | 97,90 | 1542 | 250 |  | 946 | 1743 | 2203 |
| 98,40 | 77,80 | 96,07 | 99,42 | 95,30 | 97,53 | 2404 | 3675 | 1853 | 4257 | 2517 | 2397 | 90,20 | 97,90 | 97,20 | 99,42 | 98,80 | 98,50 | 625 | 1325 | 454 | 1500 | 766 | 1003 |
| 96,40 | 95,00 | 92,90 | 97,50 | 98,30 | 97,82 | 2306 | 3650 | 1675 | 2075 | 2680 | 3711 | 93,60 | 93,75 | 98,20 | 98,27 | 96,70 | 97,30 | 653 | 1227 | 1100 | 1272 | 702 | 2446 |
| 98,00 | 88,20 | 92,80 | 98,50 | 96,10 | 96,52 | 2280 | 3625 | 4050 | 2944 | 4010 | 1762 | 94,20 | 99,57 | 99,40 | 99,67 | 98,00 | 98,20 | 606 | 1900 | 2008 | 2229 | 930 | 904 |
| 98,30 | 95,60 | 95,97 | 95,30 | 96,80 | 97,03 | 2267 | 3400 | 2342 | 3164 | 4907 | 5141 | 97,80 | 98,87 | 98,40 | 94,62 | 97,60 | 93,20 | 651 | 1846 | 1700 | 1917 | 2890 | 1738 |
| 94,70 | 97,40 | 93,93 |  | 96,00 | 97,22 | 2091 | 3092 | 2971 |  | 2459 | 2630 | 88,70 | 96,02 | 94,90 | 68,52 | 98,07 | 98,70 | 707 | 675 | 875 | 261 | 1864 | 2077 |
|  | 97,60 | 98,32 |  | 98,90 | 98,05 |  | 3056 | 2404 |  | 2820 | 2645 |  | 98,33 | 94,80 | 95,68 | 98,02 | 97,60 |  | 2070 | 1625 | 1227 | 1829 | 2055 |
|  | 99,10 | 97,80 | 97,50 | 98,40 | 98,42 |  | 2975 | 2100 | 2437 | 2669 | 4276 |  | 93,27 | 94,40 | 95,47 | 98,02 | 95,60 |  | 725 | 824 | 1463 | 699 | 1025 |
|  | 98,80 | 96,90 | 93,85 |  |  |  | 2925 | 1664 | 2157 |  |  |  | 97,75 | 96,20 | 96,25 |  |  |  | 1471 | 930 | 913 |  |  |
|  | 91,90 | 96,72 | 89,70 |  |  |  | 2925 | 3050 | 3425 |  |  |  | 98,80 | 94,80 | 77,85 |  |  |  | 2130 | 825 | 1227 |  |  |
|  | 98,60 | 94,92 | 95,97 |  |  |  | 2832 | 1975 | 1522 |  |  |  | 98,05 | 95,40 | 93,67 |  |  |  | 1063 | 1423 | 1341 |  |  |
|  | 98,90 | 98,05 |  |  |  |  | 2820 | 2645 |  |  |  |  | 97,28 | 95,10 | 91,85 |  |  |  | 1400 | 875 | 1200 |  |  |
|  | 90,70 | 95,17 | 96,30 |  |  |  | 2748 | 1808 | 2347 |  |  |  | 98,42 | 95,40 | 96,27 |  |  |  | 1037 | 800 | 750 |  |  |
|  | 98,30 | 97,82 |  |  |  |  | 2680 | 3711 |  |  |  |  | 98,00 | 95,70 |  |  |  |  | 2801 | 1125 |  |  |  |
|  | 98,40 | 98,42 |  |  |  |  | 2669 | 4276 |  |  |  |  | 99,37 | 98,40 | 39,20 |  |  |  | 1475 | 9325 | 2450 |  |  |
|  | 81,80 | 97,10 | 96,88 |  |  |  | 2575 | 2454 | 2811 |  |  |  | 96,18 | 97,90 | 94,05 |  |  |  | 922 | 475 | 124 |  |  |
|  | 97,90 | 96,07 | 94,88 |  |  |  | 2572 | 2213 | 1972 |  |  |  | 99,42 | 97,90 | 79,10 |  |  |  | 1424 | 1448 | 1052 |  |  |
|  | 95,30 | 97,53 |  |  |  |  | 2517 | 2397 |  |  |  |  | 96,92 | 98,50 | 93,92 |  |  |  | 575 | 850 | 625 |  |  |
|  | 96,00 | 97,22 |  |  |  |  | 2459 | 2630 |  |  |  |  | 98,68 |  | 96,87 |  |  |  | 1675 |  | 1423 |  |  |
|  | 98,80 | 97,65 | 83,17 |  |  |  | 2342 | 2125 | 1097 |  |  |  | 98,57 | 96,80 | 96,35 |  |  |  | 980 | 950 | 450 |  |  |
|  | 90,50 | 97,92 | 97,25 |  |  |  | 2300 | 2034 | 2034 |  |  |  | 97,72 | 96,80 |  |  |  |  | 2140 | 1550 |  |  |  |
|  | 98,10 | 96,95 | 95,18 |  |  |  | 2231 | 2775 | 3007 |  |  |  | 91,83 | 79,40 | 87,20 |  |  |  | 700 | 876 | 865 |  |  |
|  | 98,50 | 98,82 |  |  |  |  | 2173 | 2068 |  |  |  |  | 96,40 | 97,90 | 97,30 |  |  |  | 1725 | 1723 | 1723 |  |  |
|  | 97,70 | 97,68 |  |  |  |  | 2120 | 2120 |  |  |  |  | 94,22 | 96,60 | 98,17 |  |  |  | 2375 | 1360 | 1036 |  |  |
|  | | | | | | | | | | | | | | | | | | | | | | | |
| **Monocytes** | | | | | | | | | | | | | | | | | | | | | | | |
| **P-R** | | | | | | **P-MFI** | | | | | | **B-R** | | | | | | **B-MFI** | | | | | |
| HC | Group A | | | Group B | | HC | Group A | | | Group B | | HC | Group A | | | Group B | | HC | Group A | | | Grroup B | |
| 87,03 | 46,25 | 66,93 | 81,03 | 40,43 | 65,60 | 1501 | 1316 | 1431 | 1280 | 2021 | 1585 | 47,90 | 71,45 | 62,00 | 36,33 | 49,98 | 58,33 | 249 | 3219 | 295 | 373 | 1659 | 294 |
| 89,30 | 71,00 |  |  | 50,87 | 67,98 | 1823 | 2000 |  |  | 2875 | 2051 | 66,56 | 59,00 |  |  | 33,60 | 64,73 | 150 | 330 |  |  | 545 | 288 |
| 64,10 | 82,12 | 87,00 | 78,00 | 61,18 |  | 1598 | 1046 | 1807 | 1447 | 1219 |  | 48,76 | 73,37 | 65,00 | 53,00 | 50,28 |  | 638 | 317 | 337 | 280 | 282 |  |
| 82.90 | 68,93 | 40,95 | 57,03 | 51,12 | 57,50 | 2557 | 2227 | 95 | 2089 | 3431 | 2525 | 58,30 | 59,00 | 62,43 | 71,00 | 47,65 | 42,80 | 367 | 405 | 340 | 321 | 409 | 415 |
| 75.70 | 71,50 |  | 60,33 | 78,58 | 78,62 | 2193 | 39 |  | 2149 | 2579 | 2233, | 82,10 | 49,03 |  | 73,17 | 63,60 | 85,92 | 386 | 203 |  | 318 | 388 | 357 |
| 41.90 | 44,10 | 52,03 | 61,30 | 60,57 | 51,53 | 1368 | 1840 | 1300 | 682 | 2246 | 1321 | 64,80 | 47,97 | 51,73 | 42,83 | 33,07 | 45,35 | 252 | 290 | 111 | 137 | 325 | 345 |
| 69,60 | 74,08 | 23,90 | 60,97 | 90,68 | 52,25 | 1212 | 1408 | 722 | 1388 | 2290 | 1863 | 51,30 | 56,73 | 66,60 | 76,33 | 77,55 | 42,78 | 226 | 220 | 340 | 259 | 257 | 399 |
| 48,16 | 70,93 | 56,87 | 49,20 | 46,88 | 42,23 | 1409 | 1740 | 1187 | 1328 | 1193 | 2354 | 62,80 | 51,58 | 86,12 | 91,50 | 73,70 | 26,52 | 221 | 462 | 286 | 240 | 277 | 303 |
| 49,20 | 43,00 | 56,00 | 62,00 | 88,65 | 49,02 | 1497 | 1118 | 1457 | 1335 | 3506 | 2725 |  | 43,00 | 83,00 | 78,00 | 55,80 | 61,65 |  | 478 | 256 | 380 | 598 | 11 |
| 28,90 | 74,03 | 33,95 | 69,00 | 71,88 | 65,27 | 1277 | 1712 | 2465 | 1195 | 1813 | 2143 | 59,60 | 62,38 | 66,77 |  | 75,23 | 66,68 | 253 | 245 | 270 |  | 340 | 354 |
|  | 34,48 | 25,00 | 48,00 | 62,60 | 46,00 |  | 1351 | 787 | 681 | 1849 | 989 |  | 59,57 | 67,00 | 47,00 | 63,93 | 62,00 |  | 440 | 375 | 379 | 467 | 412 |
|  | 80,95 | 68,40 | 71,00 | 90,32 | 88,48 |  | 1647 | 892 | 1352 | 2201 | 2763 |  | 47,47 | 56,05 |  | 75,02 | 72,75 |  | 217 | 263 |  | 259 | 286 |
|  | 38,48 | 51,15 | 56,82 |  |  |  | 2014 | 1083 | 1186 |  |  |  | 28,87 | 40,43 | 21,18 |  |  |  | 337 | 261 | 254 |  |  |
|  | 80,10 | 46,00 | 72,00 |  |  |  | 1385 | 1080 | 2195 |  |  |  | 52,87 | 30,00 | 69,00 |  |  |  | 208 | 282 | 380 |  |  |
|  | 62,83 | 76,92 | 61,05 |  |  |  | 2411 | 1740 | 1725 |  |  |  | 59,53 | 57,72 | 73,90 |  |  |  | 238 | 335 | 305 |  |  |
|  | 50,17 | 70,07 |  |  |  |  | 2340 | 1420 |  |  |  |  | 56,00 |  |  |  |  |  | 405 |  |  |  |  |
|  | 26,70 |  |  |  |  |  | 607 |  |  |  |  |  | 18,57 |  |  |  |  |  | 278 |  |  |  |  |
|  | 71,30 |  |  |  |  |  | 2000 |  |  |  |  |  | 59,00 |  |  |  |  |  | 331 |  |  |  |  |
|  | 43,60 | 72,10 | 88,20 |  |  |  | 2000 | 1425 | 1200 |  |  |  | 58,60 | 27,10 |  |  |  |  | 417 | 222 |  |  |  |
|  | 73,00 | 62,00 | 59,00 |  |  |  | 1627 | 1457 | 1335 |  |  |  | 77,00 | 33,00 | 75,00 |  |  |  | 460 | 255 | 380 |  |  |
|  | 50,77 | 57,72 | 46,97 |  |  |  | 1974 | 1557 | 981 |  |  |  | 48,83 | 37,55 | 48,50 |  |  |  | 251 | 442 | 265 |  |  |
|  | 60,00 | 59,00 | 79,00 |  |  |  | 1875 | 1035 | 1330 |  |  |  | 46,00 | 83,00 | 33,00 |  |  |  | 335 | 282 | 207 |  |  |
|  | 65,63 |  | 80,03 |  |  |  | 1443 |  | 1398 |  |  |  | 28,73 |  | 61,77 |  |  |  | 296 |  | 350 |  |  |
|  | 81,00 | 68,00 | 65,00 |  |  |  | 1962 | 1997 | 1562 |  |  |  | 78,00 | 79,00 | 34,00 |  |  |  | 307 | 382 | 220 |  |  |
|  | 53,00 | 77,00 | 39,10 |  |  |  | 1525 | 2050 | 1700 |  |  |  | 63,00 | 79,00 |  |  |  |  | 337 | 467 |  |  |  |
|  | 52,42 | 41,03 |  |  |  |  | 2750 | 1668 |  |  |  |  | 63,58 | 82,00 |  |  |  |  | 275 | 340 |  |  |  |
|  | 41,23 | 28,37 | 55,20 |  |  |  | 1652 | 1812 | 1661 |  |  |  | 59,22 | 57,95 | 47,18 |  |  |  | 267 | 308 | 235 |  |  |
|  | 38,97 | 25,87 | 39,70 |  |  |  | 2155 | 1748 | 1862 |  |  |  | 42,67 | 38,67 | 31,23 |  |  |  | 470 | 342 | 284 |  |  |
